# Metabolomic Profile of Skeletal Muscle and Its Change Under a Mixed-Mode Exercise Intervention in Progressively Dysglycemic Subjects

**DOI:** 10.3389/fendo.2021.778442

**Published:** 2021-12-06

**Authors:** Lukasz Szczerbinski, Aleksandra Golonko, Mark Taylor, Urszula Puchta, Paulina Konopka, Adam Paszko, Anna Citko, Karol Szczerbinski, Maria Gorska, Piotr Zabielski, Agnieszka Błachnio-Zabielska, Steen Larsen, Adam Kretowski

**Affiliations:** ^1^ Department of Endocrinology, Diabetology and Internal Medicine, Medical University of Bialystok, Bialystok, Poland; ^2^ Clinical Research Centre, Medical University of Bialystok, Bialystok, Poland; ^3^ Helen Diller Family Comprehensive Cancer Center, University of California at San Francisco, San Francisco, CA, United States; ^4^ Department of Medical Biology, Medical University of Bialystok, Bialystok, Poland; ^5^ Department of Hygiene, Epidemiology and Metabolic Disorders, Medical University of Bialystok, Bialystok, Poland; ^6^ Department of Biomedical Sciences, University of Copenhagen, Copenhagen, Denmark

**Keywords:** exercise intervention, skeletal muscles, diabetes, prediabetes, metabolomics

## Abstract

Skeletal muscles play an essential role in whole-body glucose homeostasis. They are a key organ system engaged in the development of insulin resistance, and also a crucial tissue mediating the beneficial metabolic effects of physical activity. However, molecular mechanisms underlying both these processes in skeletal muscle remain unclear. The aim of our study was to compare metabolomic profiles in skeletal muscle of patients at different stages of dysglycemia, from normoglycemia through prediabetes to T2D, and its changes under a mixed-mode (strength and endurance) exercise intervention. We performed targeted metabolomics comprising several major metabolite classes, including amino acids, biogenic amines and lipid subgroups in skeletal muscles of male patients. Dysglycemic groups differed significantly at baseline in lysophosphatidylcholines, phosphatidylcholines, sphingomyelins, glutamine, ornithine, and carnosine. Following the exercise intervention, we detected significant changes in lipids and metabolites related to lipid metabolism, including in ceramides and acylcarnitines. With their larger and more significant change over the intervention and among dysglycemic groups, these findings suggest that lipid species may play a predominant role in both the pathogenesis of type 2 diabetes and its protection by exercise. Simultaneously, we demonstrated that amino acid metabolism, especially glutamate dysregulation, is correlated to the development of insulin resistance and parallels disturbances in lipid metabolites.

## Introduction

Type 2 diabetes (T2D) is one of the most common metabolic disorder. With the prognostics that by 2045 globally there will be 400 million people with diabetes, with vast majority being type 2, makes the disease one of the most emerging challenges of the healthcare systems worldwide (1). T2D is primarily caused by two main factors: insulin resistance and insufficient insulin secretion (2). Insulin resistance is defined as impaired response of cells and tissues to the insulin action, causing increased blood glucose (3).

Skeletal muscles play an essential role in the whole-body glucose homeostasis, being the main insulin-sensitive organ, along with liver and adipose tissue. In healthy individuals, skeletal muscles contribute to around 40% of total body mass and are an essential site for glucose disposal. It was shown that T2D development is associated with decreased muscle mass, altered fiber composition, mitochondrial dysfunction and ectopic accumulation of lipids in the tissue, combined with altered concentration of different lipid species, including diacylglycerol (DAG), ceramides or sphingolipids (4–9). However, it is still debated whether these changes in skeletal muscles precede the development of T2D and contribute to the etiology of the disease, or whether it is in fact a consequence of the disease (10).

Exercise is a key therapeutic tool in treatment of many diseases, including T2D, obesity or non-alcoholic fatty liver disease. Physical activity is also very effective in the prevention of metabolic diseases. It was shown that regular activity, of 150 minutes per week with the moderate to vigorous intensity, can reduce the risk for T2D by 30% (11). The effectiveness of exercise is a result of the metabolic adaptations present in almost every tissue and the inter-organ crosstalk between them (12). In this process the orchestral role is played by skeletal muscles, where multiple adaptations are observed in response to exercise, including increased insulin-dependent and -independent glucose uptake (13) or increased mitochondria biogenesis and its improved function (14, 15). This makes this tissue the biggest target of interest in the mechanistical studies of exercise effectiveness in metabolic diseases therapy.

Metabolomics gives an opportunity for simultaneous measurement of numerous metabolites – small molecules, including sugars, lipids, amino acids, amines or steroids. Over the last two decades, both targeted and untargeted metabolomics, has been used to better understand the mechanisms of T2D development and progression, but also to detect biomarkers of the disease and its complications. For example, the strong association of increased concentration of branch chain amino acids (BCAAs) and higher risk of T2D development emerged from metabolomics. This also applies to the skeletal muscle metabolomics – in the tissue where the dynamic role of the metabolites is particularly essential. Lipidomic studies, the subtype of metabolomics focused on lipid metabolites, revealed the increased concentrations of diacylglycerols (DAGs) and ceramides in muscles of subjects with insulin resistance (16, 17). Moreover, studies showed that exercise training might induce improvements in insulin sensitivity by reducing DAGs and ceramides concentration in this tissue (18). However, the role of intramuscular lipids in pathogenesis of T2D and exercise-induced adaptations in the prevention and treatment of the disease are still debated.

Thus, the aim of our study was to compare profile of metabolite concentrations in skeletal muscle of patients at different stages of dysglycemia, from normoglycemia, through prediabetes to T2D and its changes under the exercise intervention, using targeted metabolomics approach.

## Materials and Methods

### Study Population

Patients in this analysis were selected from the “Bialystok Exercise Study in Diabetes (BESD)” cohort. This study was conducted at the Department of Endocrinology, Diabetology and Internal Medicine and Clinical Research Centre of the Medical University of Bialystok. Participants of the BESD study were sedentary males at different stages of dysglycemia, living in the city of Bialystok. All subjects participated in an exercise intervention with clinical assessment before and after the intervention.

In this study, we selected subjects from the BESD cohort for whom frozen muscle tissue was available for metabolomic assay. Participants were divided according to ADA diagnostic criteria into three groups:

A. NORMOGLYCEMIA (n = 11) – subjects with normal fasting glucose and normal glucose tolerance.
B. PREDIABETES (n = 13) – subjects with impaired fasting glucose and impaired glucose tolerance
C. T2D group (n = 8) – subjects with type 2 diabetes, diagnosed within last 3-5 years, treated with metformin only as an anti-diabetic drug

Subjects within these groups were matched for age, number of performed trainings during the intervention, and changes in diet by confirming non-significant differences according to t-tests.

The study was conducted in accordance with the ethical standards of the institutional research committee and with the 1964 Helsinki declaration and its later amendments and was approved by the local ethics committee of the Medical University of Bialystok (approval number: R-I-002/469/2014). All study participants provided written informed consent.

### Exercise Intervention

Studied subjects participated in supervised three-month exercise intervention. Each training session consisted of aerobic (60-70% of an individual’s VO2max) and strength (60-75% of repetition max increased gradually by individual adjustment every three weeks) activities. The frequency of training was 3 sessions per week for 12 weeks, for a total number of trainings of 36. Each training lasted approximately 85 min, beginning with a warm-up (15 min), strength exercises (40 min - exercises involving all main groups of muscles), and finally followed by endurance exercises (30 min of biking or running on stationary equipment). The intensity was personalized and adjusted for each patient. Specifically, for the endurance activities, the target HR range corresponded to 60–70% of individual’s VO2max and for resistance exercises, 60–75% of 1 Repetition Max loads for each exercise increased gradually every three weeks. A significant feature of the exercise intervention is that it was extremely closely supervised to ensure uniformity among patients in the exercise intervention. Specifically, there was one-on-one supervision for each subject during each training session by an exercise technician to ensure no confounding differences in exercise performance among patients emerged. For this particular study, only subjects who performed at least 80% of all planned trainings were included.

### Clinical and Biochemical Analyses

Each participant underwent clinical assessment before and after the 3-month intervention, in three-visit panels: visit 1 with the oral glucose tolerance test (OGTT), visit 2 with skeletal muscle biopsy and body composition measurements and visit 3 with the maximal cardiopulmonary exercise test (CPET) performed on treadmill (Quasar Med, h/p/cosmos, Germany) using progressive Balke standard protocol for the measurement of maximal oxygen consumption (VO2max) (19). All the study visits were performed in fasting state, within 48 hours of initiating and terminating the intervention, respectively.

Muscle biopsy were obtained from the vastus lateralis muscle (VL) using a percutaneous needle with applied suction (modified Bergstrom technique) (20). Immediately after collection, samples were visually inspected and processed to remove excess blood, connective tissue and fat, then the weight of each sample was measured on a laboratory scale and after that, all of them were snap-frozen and stored in liquid nitrogen.

Body composition assays were performed with the whole-body dual energy X-ray absorptiometry (DXA), using Lunar iDXA (GE Healthcare, USA). The total amount of lean body mass (LBM), fat mass (FM) and visceral adipose tissue mass (VAT mass) were measured.

Biochemical measurements of serum triglycerides (TG), total cholesterol (TChol), high-density lipoprotein cholesterol (HDL) and low-density lipoprotein cholesterol (LDL) concentrations were done using colorimetric methods with Cobas c111 (Roche Diagnostics, Switzerland). Haemoglobin A1c (HbA1c) was measured by the high-performance liquid chromatography (HPLC) method (Bio-Rad VARIANT, Bio-Rad Laboratories, USA).

CPET was performed on treadmill (Quasar Med, h/p/cosmos, Germany), connected to the metabolic assay cart (Quark, Cosmed, Italy), using the progressive Balke standard protocol to measure maximal oxygen consumption (VO2max) (19).

### Skeletal Muscle Metabolomics

Targeted metabolomics was performed using high-resolution mass spectrometric-based AbsoluteIDQ^®^ p400 HR kit (Biocrates, Austria). This kit quantitatively measures metabolites of several classes including amino acids, biogenic amines, phosphatidylcholines, lysophosphatidylcholines, sphingomyelins, acylcarnitines, cholesteryl esters, diglycerides and triglycerides. The complete list of metabolites is presented in the **Supplementary Table 1**. Sample preparation and analysis were performed according to manufacturer specifications. In brief, tissue was homogenized using the GenoGrinder Automated Tissue Homogenizer (SPEX^®^ SamplePrep, USA) with 3mm stainless steel balls and 3uL of cold methanol per mg of tissue. Following centrifugation, 10 ul of the homogenate supernatant were added to the well-plate of the kit. A Thermo Scientific Vanquish UPLC paired with a Thermo Scientific QE-HF Mass Spectrometer was used for the analysis of the samples. The quantification of metabolite concentrations and data processing were done using the MetIDQ software, dedicated to the analysis of Biocrates metabolomics panels. The assay employed a mix of isotope-labelled internal standards, external calibration curves and quality control samples to generate quantitative results for every analyte. All values are expressed in pmol/mg of tissue.

To generate a detailed profile of ceramide content, we used a more efficient extraction and detection method since ceramides are a methodologically challenging lipid species to assay for technical reasons (21). Ceramides were measured using an ultra-high performance liquid chromatography-tandem mass spectrometry (UHPLC/MS/MS) approach according to Blachnio-Zabielska et al. (22). Briefly, samples (~20 mg) were pulverized in LN2 homogenized in a solution composed of 0.25 M sucrose, 25 mM KCl, 50 mM Tris, and 0.5 mM EDTA, pH 7.4. Following this, the internal standard solution (C15-d7-Cer, C16:0-d7-Cer, C18:0-d7-Cer, C24:0-d7-Cer, C24:1-d7-Cer, d17:1/18:1-Cer, d17:1/20:0-Cer, Avanti Polar Lipids, Alabaster, AL, USA) as well as extraction mixture (isopropanol:water:ethyl acetate, 30:10:60; v/v/v) were added to each sample. The mixture was vortexed, sonicated and centrifuged for 10 min at 4000 g (MPW 350R). The supernatant was transferred to a new vial and pellet was re-extracted with the same extraction mixture. After centrifugation, supernatants were combined and evaporated under nitrogen. Then, samples were reconstituted in LC Solvent B (2 mM ammonium formate, 0.1% formic acid in methanol. Ceramides were analyzed with the use of a Sciex QTRAP 6500 + triple quadrupole mass spectrometer, using positive ion electrospray ionization (ESI) source (except of S1P, which was analyzed in negative mode) with multiple reaction monitoring (MRM) against standard curves constructed for each compound. The chromatographic separation was performed using reverse-phase column (Zorbax SB-C8 column 2.1 × 150 mm, 1.8 μm) (Agilent, Santa Clara, CA, USA). Chromatographic separation was conducted in binary gradient using 1 mM ammonium formate, 0.1% formic acid in water as solvent A and 2 mM Ammonium formate, 0.1% formic acid in methanol as solvent B at the flow rate of 0.4 mL/min.

For the abbreviations for lipid classes metabolites, written as [class name] x:y, x describes total number of carbons and y total number of double bonds of all chains in the molecule.

### Statistical Analyses

All anthropometric and metabolomic parameters were tested for normality by the Kolmogorov-Smirnov test and outliers within each parameter, defined as points 1.5xIQR less than and greater than the first and third quartiles respectively, were removed. If parameters were not normal, they were analyzed with non-parametric methods. For normally distributed variables within a timepoint, two independent one-way ANOVAs were performed to test for differences among diagnosis groups before and after the intervention. Next, two-way ANOVAs were performed across time points and diagnoses groups to test for their interacting effects. If these tests were significant, *post-hoc* analysis was done to compare specific factor levels Sidak or Dunnet *post-hoc* tests. We present these results as “before,” which refers to an isolated one-way ANOVA among groups prior to the intervention; “after” referring to an isolated one-way ANOVA among groups following the intervention; “change” referring to the significance of the interaction term for two-way ANOVAs; and “before *vs*. after” reporting the time effect within the two-way ANOVA. P-values were corrected for total study-wise tests using the Benjamini-Hochberg procedure. This is a conservative approach to test for time and interaction effects, which may introduce a significant type 2 error burden. However, we considered it more important to be confident in positive effects (low type 1 error) than in negative effects (low type 2 error). For non-normally distributed variables, non-parametric Kruskall-Wallis tests were used to determine whether the intervention or differences among diagnosis groups were significant. Furthermore, for metabolomics, we included only metabolites with >50% of values above the limit of detection (>LOD) across samples.

## Results

### Clinical Parameters

Characteristics of study participants stratified into glycemic diagnosis groups are presented in **Table 1**. As expected, we found significant differences in most of the studied metabolic and anthropometric parameters, including HbA1c, both fasting and 2-hour glucose, HOMA-b, HOMA-IR, total and visceral fat mass, as well as exercise capacity (VO2max). Interestingly for parameters of insulin sensitivity (HOMA-IR and Matsuda index), the most advanced insulin resistance appeared not in T2D but in prediabetic patients. This is likely because all diabetic patients were metformin-only treated with well controlled diabetes (<6.5% HbA1c). The results of the clinical effects of the exercise are presented in **Supplementary Table 2**.

**Table 1:** Clinical characteristic of studied groups before the initiation of the exercise intervention.

Parameter	NORMOGLYCEMIA	PREDIABETES	T2D	p-value
**Age (years)**	48.5 (43.7-53.3)	48.6 (43.4-53.7)	50.1 (45.5-51.4)	0.185
**Weight (kg)**	83.8 (77.99-89.61)	101.19 (94.39-108)	88.89 (79.36-98.42)	**0.001**
**HbA1c (%)**	5.26 (5.04-5.47)	5.69 (5.48-5.91)	5.99 (5.58-6.4)	**0.001**
**Fasting Glucose (mg/dl)**	95.73 (91.04-100.41)	119.38 (112.13-126.64)	124.13 (113.78-134.47)	**<0.001**
**2-h Glucose (mg/dl)**	92.73 (75.59-109.86)	161.23 (147.61-174.85)	233.25 (180.63-285.87)	**<0.001**
**Fasting Insulin (μU/mL)**	9.89 (7.41-12.37)	23.37 (16.78-29.97)	12.34 (8.32-16.35)	**<0.001**
**HOMA-b (%)**	111.68 (82.67-140.7)	153.57 (110.13-197)	77.17 (41.91-112.43)	**0.017**
**HOMA-IR**	2.35 (1.73-2.96)	6.94 (4.86-9.03)	3.77 (2.53-5.01)	**<0.001**
**BMI (kg/m^2^)**	25.69 (24.19-27.2)	32.88 (30.41-35.34)	29.68 (26.62-32.74)	**<0.001**
**Fat mass (kg)**	23.37 (19.05-27.7)	35.53 (31.89-39.17)	27.54 (21.32-33.76)	**<0.001**
**Lean mass (kg)**	57.15 (53.57-60.73)	62.24 (58.24-66.25)	58.27 (53.26-63.29)	0.115
**VAT mass (kg)**	1.35 (0.9-1.8)	2.86 (2.3-3.41)	2.45 (1.57-3.33)	**0.001**
**Total Chol. (mg/dl)**	214.82 (187.42-242.22)	209.69 (193.67-225.72)	195.75 (158.33-233.17)	0.530
**TG (mg/dl)**	103.55 (70.88-136.21)	152.85 (123.28-182.41)	137.25 (110.96-163.54)	**0.040**
**HDL (mg/dl)**	61.36 (51.48-71.25)	52 (43.88-60.12)	56.25 (48.84-63.66)	0.229
**LDL (mg/dl)**	142.05 (115.76-168.34)	139.99 (124.75-155.22)	124.78 (89.64-159.92)	0.528
**VO2max (ml/kg/min)**	33.69 (29.76-37.62)	27.56 (26.07-29.06)	26.86 (23.34-30.38)	**0.002**
**Matsuda index**	4.73 (3.69-5.77)	1.64 (1.08-2.19)	2.95 (1.5-4.4)	**<0.001**

Presented are mean values with 95% confidence intervals (95%CI). Bold are significant p-values (p < 0.05).

### Metabolomics Results

#### Amino Acids

**Figure 1:**
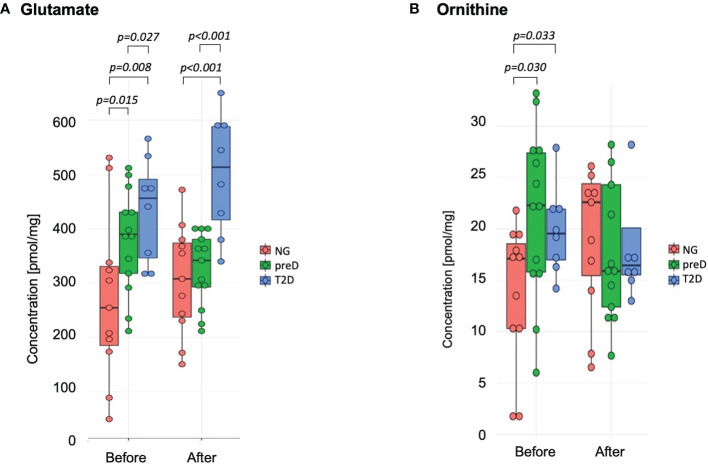
Amino acids with significant differences among diagnosis groups – normoglycemia (NG), prediabetes (preD) and type 2 diabetes (T2D), before and after 3 months of exercise intervention. **(A)** Glutamate; **(B)** Ornithine. Mean and standard errors are presented.

Among amino acids exceeding their respective LODs, we found significant differences among groups before the intervention for glutamate and ornithine (see **Figure 1**). We found that glutamate concentration was highest in the T2D group and lowest in the normoglycemic group (p=0.015). For ornithine we found that normoglycemic subjects had significantly lower concentrations, compared to the two remaining groups (p=0.30). After the exercise intervention, these differences remained significant for glutamate but not ornithine (p=0.948) because of the increase in the normoglycemic group (**Figure 1**).

When we tested for the effect of the exercise in general, comparing concentrations of metabolites before and after the intervention, we found significant increase in aspartate concentration (12.31 *vs*. 21.28; p<0.001) with no differences in response between groups (p=0.081).

We also tested correlations of amino acids with clinical parameters and we found significant correlation of glutamate with 2-h glucose concentration (r^2 =^ 0.59; p<0.001) and fasting glucose (r^2 =^ 0.45; p<0.01) for baseline measurements.

#### Biogenic Amines

**Figure 2:**
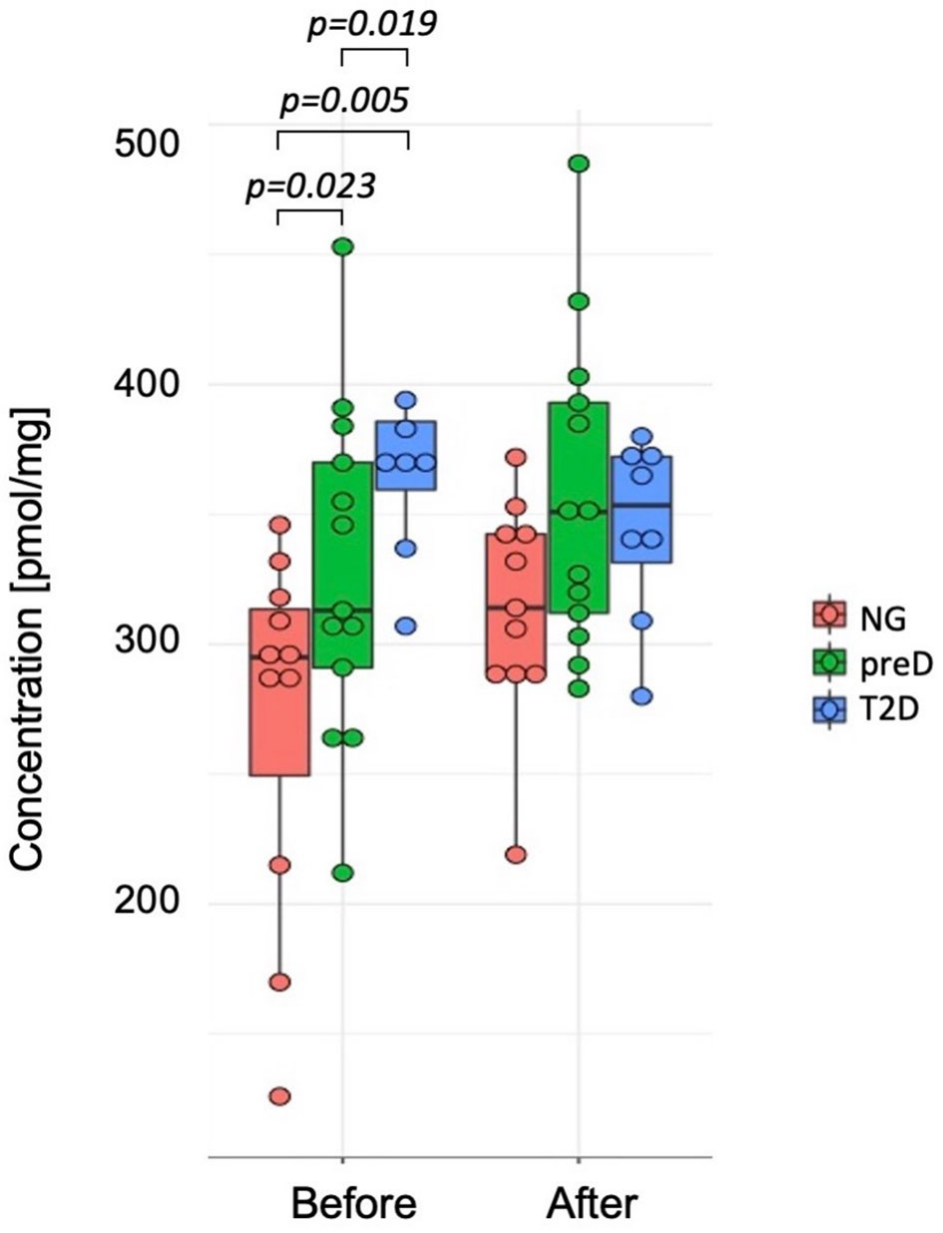
Differences in carnosine concentrations among diagnosis groups – normoglycemia (NG), prediabetes (preD) and type 2 diabetes (T2D), before and after 3 months of exercise intervention. Mean and standard errors are presented.

Among biogenic amines, we found significant differences between groups before the intervention for carnosine where the highest concentration was detected in T2D group and the lowest in normoglycemic group (p=0.005). After the exercise intervention, these differences were not significant (p=0.107) because of the differences in response between normoglycemics and two remaining groups, with the p-value for differences in change (change= concentration after – concentration before) equal 0.038. (see **Figure 2**).

The exercise intervention significantly increased concentrations of histamine (1.58 *vs*. 1.87; p=0.011) and putrescine (0.46 *vs*. 0.57; p=0.026).

We also found significant correlations between baseline carnosine and fasting glucose (r2 = 0.36; p<0.05), 2-h glucose (r2 = 0.61; p<0.001), HbA1c (r2 = 0.37; p<0.05), fat mass (r2 = 0.39; p<0.05) and visceral fat mass (r2 = 0.43; p<0.05), VO2max (r2=-0.47; p<0.01) and Matsuda index (r2=-0.4; p<0.05). However, we did not detect significant correlation among changes in biogenic amines and clinical parameters under the exercise intervention.

#### Acylcarnitines

We did not uncover any significant differences in measured acylcarnitines (ACs) concentrations between studied groups. However, we did detect significant changes in concentration of number of ACs under the exercise intervention (**Table 2**) with no differences in the response between groups. Concentrations of ACs in each of studied groups, before and after the intervention, are presented in **Supplementary Table 3**.

**Table 2:** Detected significant differences in concentrations of measured ACs before and after the exercise intervention in all studied patients.

Full name	Short name	Before	After	p-value
**Propionylcarnitine **	AC 3:0	1.34 (0.96-1.71)	1.07 (0.86-1.28)	**0.003**
**Valerylcarnitine **	AC 5:0	0.57 (0.32-0.83)	0.39 (0.22-0.56)	**0.003**
**Hydroxyvalerylcarnitine **	AC 5:0-OH	0.15 (0.06-0.24)	0.09 (0.05-0.14)	**0.001**
**Malonylcarnitine **	AC 3:0-DC	0.02 (0.01-0.03)	0.03 (0.02-0.04)	**0.016**
**Hydroxyhexanoylcarnitine **	AC 6:0-OH	0.08 (0.05-0.11)	0.11 (0.07-0.15)	**0.001**
**Decanoylcarnitine **	AC 10:0	0.28 (0.17-0.4)	0.39 (0.2-0.58)	**0.022**
**Decenoylcarnitine **	AC 10:1	0.05 (0.04-0.07)	0.07 (0.04-0.1)	**0.023**
**Dodecanoylcarnitine **	AC 12:0	0.9 (0.46-1.34)	1.36 (0.66-2.07)	**<0.001**
**Dodecenoylcarnitine **	AC 12:1	0.25 (0.17-0.34)	0.33 (0.24-0.42)	**0.010**
**Tridecanoylcarnitine **	AC 13:0	0.04 (0.02-0.05)	0.05 (0.02-0.07)	**0.007**
**Tetradecanoylcarnitine **	AC 14:0	1.65 (0.97-2.32)	2.47 (1.46-3.47)	**0.001**
**Tetradecenoylcarnitine **	AC 14:1	1.61 (0.94-2.27)	2.24 (1.51-2.98)	**0.003**
**Tetradecadienoylcarnitine **	AC 14:2	0.27 (0.15-0.39)	0.37 (0.25-0.49)	**0.006**
**Pentadecanoylcarnitine **	AC 15:0	0.19 (0.11-0.27)	0.25 (0.15-0.35)	**0.017**
**Hexadecanoylcarnitine **	AC 16:0	7.83 (4.83-10.83)	11.43 (6.96-15.9)	**0.003**
**Hexadecenoylcarnitine **	AC 16:1	2.55 (1.46-3.63)	3.92 (2.32-5.52)	**0.001**
**Hexadecadienoylcarnitine **	AC 16:2	0.37 (0.16-0.57)	0.54 (0.33-0.75)	**0.001**
**Heptadecanoylcarnitine **	AC 17:0	0.18 (0.11-0.26)	0.26 (0.16-0.36)	**0.003**
**Octadecenoylcarnitine **	AC 18:1	24.86 (13.75-35.97)	37.99 (20.34-55.63)	**0.006**
**Octadecadienylcarnitine **	AC 18:2	6.86 (3.37-10.34)	10.08 (5.44-14.72)	**0.016**

Presented are mean values with 95% confidence intervals (95%CI). Bold are significant p-values (p < 0.05).

#### Lysophosphatidylcholines and Phosphatidylcholines

Among lysophosphatidylcholines, we found significant baseline differences between studied groups before the initiation of the exercise intervention (see **Table 3**). These differences consisted of highest concentrations for normoglycemic and lowest for T2D subjects. Interestingly, most of these diagnosis-specific differences were alleviated after the intervention, except LPC-O(16:1). However, only LPC (22:5) significantly changed under the intervention, showing a significant decrease in concentration.

**Table 3:** Detected significant differences in measured metabolites from LPC and PC categories, between the experimental groups.

Metabolite	NORMOGLYCEMIA		PREDIABETES		T2D		p-value			
	Before	After	Before	After	Before	After	Before	After	Change	Before *vs*. After
**Lysophosphatidylcholines**										
LPC (15:0)	0.21 (0.05-0.38)	0.18 (0.12-0.25)	0.1 (0.07-0.13)	0.13 (0.08-0.17)	0.04 (0.02-0.06)	0.05 (0.04-0.06)	***0.011^***	0.072	0.532	0.947
LPC (16:0)	3.1 (0.89-5.3)	2.21 (1.47-2.94)	1.99 (1.55-2.43)	2.3 (1.39-3.21)	1.22 (0.93-1.5)	1.41 (1.04-1.77)	***0.0076***	0.224	0.612^	0.706
LPC (20:4)	2.18 (1.51-2.86)	1.83 (1.41-2.24)	1.32 (1.07-1.57)	1.41 (1.18-1.64)	2.37 (1.8-2.93)	1.92 (1.43-2.41)	***0.007***	0.064	0.055	0.062
LPC (22:5)	0.11 (0.08-0.14)	0.09 (0.07-0.11)	0.07 (0.06-0.08)	0.06 (0.06-0.08)	0.11 (0.09-0.13)	0.08 (0.07-0.09)	***0.018***	0.115	0.100	***0.016***
LPC-O (16:1)	3.02 (0.85-5.2)	2.15 (0.91-3.39)	1.43 (0.95-1.91)	1.38 (0.83-1.94)	0.68 (0.57-0.8)	0.52 (0.44-0.61)	***0.009^***	***0.030***	0.444	0.229
**Phosphatidylcholines**										
PC (30:0)	2.68 (1.75-3.61)	2.85 (2.12-3.59)	2.1 (1.65-2.55)	2.43 (1.65-3.22)	1.5 (1.12-1.88)	1.37 (1.08-1.67)	***0.044***	***0.010***	0.549	0.436
PC (30:1)	0.51 (0.35-0.67)	0.55 (0.39-0.71)	0.49 (0.37-0.6)	0.62 (0.36-0.87)	0.28 (0.22-0.34)	0.23 (0.15-0.31)	***0.031***	***0.025***	0.412	0.308
PC (30:2)	0.12 (0.08-0.17)	0.11 (0.08-0.14)	0.1 (0.07-0.13)	0.15 (0.1-0.2)	0.06 (0.04-0.07)	0.06 (0.03-0.1)	***0.039***	***0.016***	0.047	0.082
PC (31:0)	0.66 (0.43-0.88)	0.72 (0.47-0.98)	0.58 (0.45-0.72)	0.61 (0.41-0.82)	0.3 (0.22-0.37)	0.29 (0.22-0.36)	***0.011***	***0.013***	0.883	0.571
PC (31:1)	0.07 (0.05-0.1)	0.08 (0.05-0.11)	0.07 (0.05-0.08)	0.07 (0.05-0.1)	0.04 (0.03-0.05)	0.03 (0.02-0.04)	***0.017***	***0.017***	0.795	0.503
PC (32:2)	2.17 (1.47-2.88)	2.63 (2.05-3.22)	2.09 (1.68-2.51)	2.73 (2.05-3.42)	1.25 (1.04-1.46)	1.32 (1.01-1.63)	***0.029***	***0.004***	0.428	***0.022***
PC (32:3)	0.47 (0.33-0.6)	0.6 (0.4-0.79)	0.5 (0.39-0.61)	0.6 (0.41-0.79)	0.26 (0.22-0.3)	0.28 (0.2-0.35)	***0.008***	***0.021***	0.635	0.052
PC (32:6)	0.05 (0.04-0.07)	0.09 (0.06-0.11)	0.09 (0.07-0.11)	0.1 (0.07-0.12)	0.04 (0.02-0.07)	0.07 (0.04-0.1)	***0.001***	0.227	0.381	***0.030***
PC (33:1)	1.12 (0.69-1.54)	1.29 (0.71-1.86)	1.02 (0.81-1.23)	1.04 (0.77-1.31)	0.6 (0.45-0.75)	0.53 (0.42-0.63)	***0.046***	***0.030***	0.725	0.699
PC (33:2)	1.42 (0.92-1.92)	1.77 (1.19-2.34)	1.34 (1.04-1.64)	1.61 (1.21-2)	0.69 (0.53-0.86)	0.77 (0.61-0.93)	***0.018***	***0.005***	0.707	0.070
PC (33:4)	0.08 (0.03-0.12)	0.08 (0.06-0.1)	0.06 (0.05-0.07)	0.07 (0.05-0.1)	0.04 (0.03-0.05)	0.04 (0.03-0.05)	***0.008^***	***0.070***	0.810	0.455
PC (35:2)	3.75 (2.18-5.31)	4.28 (2.81-5.75)	3.26 (2.5-4.02)	3.57 (2.77-4.37)	1.84 (1.43-2.26)	1.82 (1.51-2.12)	***0.047***	***0.007***	0.858	0.455
PC (36:2)	70.6 (32.6-108.5)	75.76 (50.0-101.4)	56.15 (41.32-70.98)	56.9 (42.34-71.46)	36.21 (29.16-43.27)	33.48 (28.08-38.87)	***0.025^***	***0.009***	0.928	0.863
PC (36:3)	44.42 (24.77-64.06)	49.95 (37.08-62.81)	36.83 (29.71-43.95)	41.81 (32.85-50.76)	24.55 (20.96-28.14)	24.16 (21.06-27.26)	***0.003^***	***0.002***	0.835	0.366
PC (37:2)	0.2 (0.08-0.32)	0.2 (0.12-0.28)	0.14 (0.1-0.18)	0.14 (0.1-0.17)	0.08 (0.06-0.11)	0.08 (0.07-0.1)	***0.015^***	***0.013***	0.997	0.981
PC (37:3)	0.4 (0.17-0.62)	0.42 (0.25-0.59)	0.31 (0.22-0.39)	0.32 (0.25-0.4)	0.18 (0.13-0.22)	0.17 (0.13-0.2)	***0.008^***	***0.014***	0.963	0.782
PC (37:6)	0.15 (0.06-0.36)	0.13 (0.04-0.21)	0.1 (0.04-0.16)	0.12 (0.05-0.2)	0.02 (0.02-0.06)	0.05 (0.02-0.09)	***0.008^***	0.325	0.733	0.722
PC (38:3)	1.88 (0.69-3.07)	1.92 (1.05-2.8)	1.36 (0.92-1.79)	1.33 (0.79-1.87)	0.8 (0.55-1.04)	0.52 (0.24-0.81)	***0.036^***	***0.016***	0.884	0.798
PC (39:6)	0.07 (0.02-0.12)	0.07 (0.04-0.11)	0.06 (0.05-0.07)	0.06 (0.04-0.08)	0.04 (0.02-0.05)	0.04 (0.03-0.05)	***0.014^***	0.158	0.944	0.666
PC-O (32:1)	0.57 (0.24-0.9)	0.54 (0.24-0.85)	0.55 (0.4-0.69)	0.46 (0.26-0.65)	0.32 (0.25-0.39)	0.23 (0.16-0.3)	***0.011^***	0.153	0.915	0.392
PC-O (35:4)	0.06 (0.03-0.1)	0.06 (0.04-0.09)	0.05 (0.04-0.06)	0.05 (0.03-0.07)	0.03 (0.03-0.04)	0.02 (0.02-0.03)	***0.006^***	***0.013***	0.922	0.605
PC-O (36:5)	38.36 (22.08-54.65)	32.92 (19.59-46.24)	21.2 (15.64-26.76)	20.67 (15.73-25.62)	20.93 (15.66-26.19)	15.18 (11.84-18.51)	***0.028***	***0.020***	0.726	0.264
PC-O (36:6)	3.47 (0.59-6.35)	2.84 (0.62-5.06)	0.86 (0.02-1.75)	0.58 (0.02-1.13)	1.69 (0.84-2.53)	1.05 (0.1-2.01)	***0.035^***	***0.049***	0.274	0.130
PC-O (38:6)	14.99 (8.35-21.63)	13.11 (7.6-18.61)	8.39 (6.04-10.75)	8.16 (6.27-10.06)	8.37 (6.27-10.47)	5.78 (4.42-7.13)	***0.040***	0.050^	0.745	0.291

Presented are mean values with 95% confidence intervals (95%CI). Three independent ANOVAs were implemented to test for “before” differences among diagnosis groups prior to the intervention; “after” differences following the intervention; “change” reporting the interacting effect of a two-way ANOVA on time x diagnosis group; and “before vs. after” reporting the time effect within the two-way ANOVA. ^ - non-parametric Kruskal-Wallis ANOVA. Bold are significant p-values (p < 0.05).

Similar trends were observed for phosphatidylcholines for which normoglycemics had the highest and T2D patients had the lowest concentrations. However, in contrast to lysophosphatidylcholines, most of these differences remained significant after the intervention. Among all measured phosphatidylcholines, only for two, PC(32:2) and PC(32:6), were significantly altered by the exercise intervention, yet still did not overcome these pre-existing baseline differences among groups (see **Table 3**).

We also found significant correlations between LPC and PC concentrations and clinical parameters. Specifically, we found a strong negative correlation among baseline concentrations of LPCs and PCs with 2-hour glucose concertation. In particular, the strongest correlation was observed for PC-O (44:4) (r2=-0.72; p<0.001) and visceral fat mass, and again with the strongest correlation for PC-O (44:4) (r2=-0.48; p<0.01).

#### Sphingomyelins

**Figure 3:**
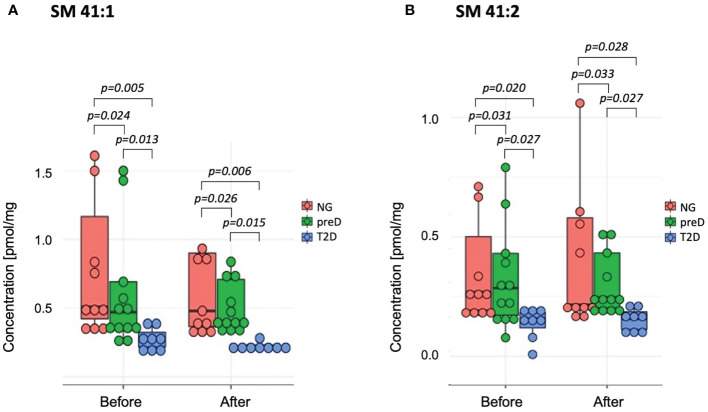
Sphingomyelins with significant differences among diagnosis groups – normoglycemia (NG), prediabetes (preD) and type 2 diabetes (T2D), before and after 3 months of exercise intervention. **(A)** SM 41:1; **(B)** SM 41:2. Mean and standard errors are presented.

Among sphingomyelins, we found significant baseline differences among groups before the intervention for two sphingomyelin species (SM 41:1 and SM 41:2 – see **Figure 3**), where the highest concentration was detected in the normoglycemic group and the lowest in the T2D group (p=0.005 for SM 41:1 and p=0.02 for SM 41:2). After the exercise intervention, these differences remained significant (p=0.006 for SM 41:1 and p=0.028 for SM 41:2).

The exercise intervention significantly increased concentrations only of SM 34:2 (1.29 *vs*. 1.55; p=0.008). We also found significant correlations between baseline concentrations of selected SMs and 2-hour glucose and HDL-cholesterol concentrations (see **Table 4**). However, we failed to detect any significant correlations of changes in SMs and clinical parameters under exercise intervention.

**Table 4:** Correlations between clinical parameters and level of sphingomyelins before the intervention.

	2-hour glucose	HDL
**SM32:1**	-0.24	0.51**
**SM32:2**	-0.05	0.55**
**SM33:1**	-0.23	0.55**
**SM34:1**	-0.11	0.42*
**SM34:2**	-0.1	0.44*
**SM35:1**	-0.27	0.42*
**SM37:1**	-0.41*	0.45**
**SM38:2**	-0.16	0.41*
**SM39:1**	-0.47**	0.45*
**SM40:2**	-0.34	0.41*
**SM41:1**	-0.53**	0.16
**SM41:2**	-0.41*	0.17
**SM42:1**	-0.42*	0.34
**SM42:3**	-0.31	0.35*

Pearson correlation coefficients (r) are presented. *p <0.05; **p < 0.01.

#### Ceramides

We did not find any significant differences in ceramide (Cer) concentrations between diagnostic groups (including total ceramide content), either before the intervention or in response to the intervention. However, we did detect significant differences in the exercise effect. Comparing samples before and after the intervention from all subjects revealed significant decreases in nearly all of the measured ceramides (**Table 5**). Concentrations of ceramides in each of studied groups, before and after the intervention, are presented in **Supplementary Table 4**. We did not find any significant correlations between ceramide content and clinical parameters.

**Table 5:** Detected significant differences in concentrations of measured ceramides before and after the exercise intervention in all studied patients.

Metabolite	Before	After	p-value
**Sph**	0.6 (0.47-0.72)	0.49 (0.41-0.56)	<0.0001
**SPA**	0.09 (0.07-0.11)	0.06 (0.05-0.08)	<0.0001
**C16:0-Cer**	0.57 (0.44-0.69)	0.46 (0.38-0.54)	0.002
**C18:1-Cer**	0.05 (0.03-0.07)	0.04 (0.02-0.05)	0.017
**C18:0-Cer**	2.16 (1.85-2.47)	1.65 (1.4-1.89)	<0.0001
**C20:0-Cer**	0.07 (0.06-0.09)	0.06 (0.05-0.07)	<0.0001
**C24:1-Cer**	2.67 (2.29-3.04)	2.32 (1.88-2.76)	0.010
**C24:0-Cer**	1.4 (1.06-1.73)	1.15 (0.96-1.33)	0.002
**Total Cer**	7.91 (6.85-8.97)	6.6 (5.84-7.36)	<0.0001

Presented are mean values with 95% confidence intervals (95%CI).

#### Cholesteryl Esters, Diglycerides, Triglycerides

We did not detect any significant differences among diagnostic groups in total intramuscular triglycerides content nor in the three remaining categories of metabolites: cholesteryl esters, diglyceride and triglycerides.

## Discussion

In our study we show significant differences in skeletal muscle metabolite profiles between normoglycemic, prediabetic and type 2 diabetic patients, representing progressive stages of dysglycemia development. What is important, these metabolites represent different chemical classes, like amino acids, acylcarnitines, phosphatidylcholines, sphingomyelins and ceramides. Moreover, similar classes of metabolites are affected by the exercise intervention, which clearly shows that molecular differences in skeletal muscles during the development of T2D are also a target to the mechanisms responsible of the beneficial metabolic effects of exercise.

### Phosphatidylcholines and Lysophosphatidylcholines

We found especially abundant differences in phosphatidylcholines and their derivates - lysophosphatidylcholines between three studied groups, before the initiation of the exercise. We show that with the progression of dysglycemia, the concentration of muscle LPCs and PC is decreasing. But interestingly, the exercise intervention did not alleviate these differences, as for vast majority of species, the differences remained significant after the intervention. Clinical studies have shown that decreases in PUFA levels in skeletal muscle phospholipids, including PCs and LPCs, are associated with lower insulin sensitivity (23, 24). However, despite the relationship between phospholipid levels and insulin sensitivity, it has not been established whether these changes are a cause or a consequence of insulin resistance (25). A recent study, by Ferrara et al., showed that obesity is associated with decreases in various species of lysophospholipids (26). Moreover, this study showed in mice, that skeletal muscle–specific inhibition of lysophosphatidylcholine acyltransferase 3 (LPCAT3), an enzyme involved in phospholipid transacylation (Lands cycle), enhances insulin signalling at the level of insulin receptor to improve skeletal muscle insulin sensitivity (26). Phospholipids can both influence the regulation of insulin secretion in the cells of the pancreas, but also participate in the action of insulin in muscle tissue, adipose tissue and intracellularly by affecting gene expression or mitochondrial function (25). Interestingly, Le Petit-Thévenin et al. have shown that insulin deficiency impaired primarily the acylation of phosphatidylcholine but not phosphatidylethanolamine in T2D rats (27). In skeletal muscle tissue, an inverse relationship was observed between the ratio of phosphatidylcholine to phosphatidylethylamine and insulin sensitivity in the group of training, diabetic adults and obese patients. However, the ratio did not change in response to physical effort (28). This is in line with the results of our research, which show significant differences between the groups, but without the impact of exercise. However, in the research of Lee et. al., it has been proven that exercise among overweight people causes enhanced levels of PC by 21%, PE by 42%, and reduced PC: PE ratio by 16%, while improving insulin sensitivity by 33% (29). In our study, for the majority of measured LPCs and PCs, we did not detect significant changes in concentration under the exercise intervention, although this does not imply that no influence exists since this study may be underpowered to detect subtle effects. We hypothesize that one of the main reasons might be a power issue to detect subtle improvements, especially if they are observed only in dysglycemic groups who tend to increase their concentrations towards normoglycemic group. To prove that, observation on bigger studied groups should be performed.

### Acylcarnitines

Interestingly, as opposed to PCs and LPCs, for measured acylcarnitines (ACs) we found a significant effect of exercise in all three studied groups, but no differences either before or after the intervention between groups were detected. Most of carnitine occurs in skeletal muscle, where it is responsible for translocating long chain (LC) fatty acids (FAs) into the mitochondrial matrix and then for incorporation into β-oxidation cycle. This is due to the fact that mitochondria are impermeable to acyl-CoA but not to fatty ACs, therefore carnitine and carnitine palmitoyltransferase are essential for translocation of LC FAs to mitochondria in skeletal muscles for β -oxidation (30). ACs are organic compounds containing a fatty acid with a carboxylic acid attached to carnitine through an ester bond. The nature of ACs is amphipathic, which means that they can accumulate in the cell membrane and affect its properties (31). Many studies have described a negative effect of LC ACs on insulin sensitivity. In the muscle ACs accumulation model (Cpt2^Sk -/-^ mice) by deleting carnitine palmitoyltransferase (CPT2), an approximately 20-fold increase in LC ACs was observed. CPT2 knockout mice was resistance to weight gain, glucose intolerance, insulin resistance, and impairments in insulin-induced Act phosphorylation during following a high-fat diet. It therefore appears that diet-induced insulin resistance is influenced by impaired flux fatty acids through mitochondrial β-oxidation. In new paradigm published by Koves in 2008 (32) suggest that β-oxidation of fatty acids exceeding the capacity of the tricarboxylic acid cycle yields incomplete fat oxidation and mitochondrial distress, obligatory events in the pathogenesis of insulin resistance (33). In our study, we did not observe significant differences in ACs concentrations between the groups, but many of them changed due to exercise. This is in line with other studies, which has shown, that in muscle tissue, several short, medium, and long-chain acylcarnitines are increased in muscle 24 hours after the last exercise session of the 6-month training intervention (34). There is evidence that disturbances in the transport of long-chain acylcarnitine to the mitochondrial matrix are one of the pathophysiological mechanisms of T2D development (35). In a four-year cohort study of 251 T2D patients, it was observed that the acylcarnitines profile was significantly associated with a higher risk of T2D. In our study, even with a lack of differences between studied groups, the effect of exercise shows that acylcarnitines and their role in lipid metabolism might be an interesting target for further studies on novel diabetes pharmacotherapy targets.

### Ceramides

We found no significant difference in ceramides content among groups, but we did detect a strong effect of exercise in all three groups for nearly all assayed ceramides, where the intervention decreased their concentration. Ceramides are a group of lipids, forming a part of the sphingolipid family. They are widely considered to be the most toxic lipids that contribute to catabolism and regulate cell growth and apoptosis, which has important implications for obesity and the consequences of T2D and cardiovascular disease. In previous studies, it was observed that the content of ceramides in skeletal muscles, especially C16- and C18-ceramide (36, 37) is inversely related to insulin sensitivity. Acute elevation of plasma FFAs results in muscle ceramide accumulation, and the increase in muscle ceramide is related to a concurrent decrease in insulin sensitivity (38). Although no differences were detected in ceramide content, we found a strong effect of exercise in all three groups especially in the context of insulin resistance C16- and C18-ceramide species, which decreased under the intervention. Similar findings were obtained in studies that found that both interval training and moderate-intensity continuous training in obese patients resulted in a decrease in the concentration of ceramides in the muscles, with no effect on diacylglycerol (39). Currently, there are conflicting results regarding the involvement of skeletal muscle ceramide accumulation in early stages of type 2 diabetes development (40) and our study contributes significantly to this open question by suggesting that in overweight/obese subjects with early metabolic disturbance, as well as patients with early stage type 2 diabetes treated with metformin, ceramides may have limited involvement in the pathogenesis of dysglycemia. However, the strongly beneficial effects of exercise on ceramide content confirms the importance of ceramides pathway as a crucial piece of exercise-induced metabolic improvements mechanisms.

### Sphingomyelins

Sphingomyelins are a type of sphingolipids, located mostly in the outer layer of the plasma membrane. When ceramide is transferred from the ER to the Golgi apparatus, complex sphingolipids are formed including sphingomyelin, lactosyl ceramides, sphingosine and sphingosine-1-phosphate. About 70% of SM is found in lipid-protein rafts in cell membranes. It has been shown that inhibition of *de novo* sphingolipid synthesis increases insulin sensitivity, and reducing their content in plasma membranes may have a significant impact on insulin signaling (41). SM accumulation prevents ceramide buildup and leads to improved insulin sensitivity in muscle cells (42). Straczkowski et al. demonstrated that obese individuals with impaired glucose tolerance showed increased muscle ceramide content and lower muscle SM compared with obese individuals with normal glucose tolerance (16). This is in line with the results of our study, where we found a gradual decrease of skeletal muscle SM concentration as dysglycemia stage advanced.

### Biogenic Amines

We observed that subjects with T2D have a higher carnosine concentration than two remaining groups, and an increase in the concentration of this dipeptide after exercise, an effect mainly confined to the normoglycemic group. Moreover, carnosine concentration in muscle was positively correlated with and fasting and 2-hour glucose, HbA1c, total and visceral fat mass, VO2max and Matsuda index. Carnosine is a dipeptide composed of two amino acids: histidine and β-alanine. Studies have shown that it can contribute to defense against tissue oxidative stress by directly interacting with reactive oxygen species (43) and superoxide radicals (44). Furthermore, higher carnosine content in human skeletal muscle have been positively associated with insulin resistance, HDL-cholesterol and atherogenic index in physically inactive middle-aged men (45). Increased muscle carnosine content may reflect the action of a compensatory mechanism to prevent cell damage in states of impaired glucose tolerance. Our results are in line with these studies which show that muscle carnosine content gradually increased with obesity and progressive glucose intolerance [for example, see (46)] More specifically, the concentration of carnosine in muscles can change dynamically and increases under the influence of exercise, which was observed both in this study and in Nordsborg et al. (47). However, exercise intervention studies show differing magnitudes of carnosine change, depending on the training mode as summarized by Derave et al. (48). Some studies report that the carnosine content of the vastus lateralis dramatically increased after 8 weeks of endurance training (49). One possible mechanisms of the increased content of carnosine after exercise, might be a compensatory antioxidant response to exercise-induced hypoxia and acidosis (48). This could explain changes in the normoglycemic group of our study, suggesting that similar mechanisms might be impaired in patients with dysglycemia.

### Cholesteryl Esters, Diglyceride, and Triglycerides

It is important to note that in our study we did not detect significant effects of dysglycemia nor of exercise on other metabolically important classes of lipids, including diglycerides, triglycerides and cholesteryl esters. However, this does not necessarily eliminate their role in the pathogenesis of T2D development. We hypothesize that this might be driven by our experimental design in which we did not measure the total content of TGs and DAGs but selected species. It may be that the bulk content of these lipid classes is more important than any single molecular species, especially since the total content of both these lipids is made by many different molecules, differencing in carbon chain length and bond saturation. Additionally, body composition appears to have a significant influence on the TG and DAG content in skeletal muscle. Previously, it was shown that weight loss among obese women with T2D resulted in a marked reduction in intracellular triglycerides, but no significant difference was observed from diglycerides (50, 51). In our study, groups were specifically differentiated by the dysglycemia status, and not body composition. Although we found significant differences in BMI and fat tissue content between studied groups, our normoglycemic group was not synonymous with normoweight subjects. Specifically, mean BMIs for both normoglycemia and T2D groups were within overweight range. Similar observations were made for fat tissue content. Several explanations have been proposed to explain the lack of observed differences among groups, but a definitive resolution will require further investigation.

### Glutamate

Among all the measured amino acids, we found that glutamate concentration was highest in the T2D group and lowest in the normoglycemic group. Glutamate, an amino acid which plays a crucial role in transamination reactions, is delivered to skeletal muscle *via* three main mechanisms: uptake from the circulation, intracellular protein degradation and by transamination of the branched chain amino acids (BCAAs), leucine, isoleucine and valine (52). This last mechanism requires a-ketoglutarate as a substrate to produce a-ketoacids and glutamate in the presence of the enzyme BCAA aminotransaminase. This amino acid is involved in several metabolic pathways in skeletal muscles, one of which is the glutathione synthesis pathway. Glutathione is an important protein in modulating levels of reactive oxygen species (ROS) and is involved in the cell’s protective oxidative stress response. Studies have shown that mitochondrial ROS release is higher in patients with type 2 diabetes, and may be a mechanism of insulin resistance (53). This might explain the observed higher concentrations of glutamate in T2D patients in our study, suggesting a chronic compensatory increase of this amino acid to protect against ROS, by increasing concentration of glutathione, in early stages of T2D development. Additionally, it is well-established that higher plasma BCAA concentration is observed in T2D patients, and that it is strongly associated with insulin resistance and is predictive of incident diabetes (54). In humans, the majority of BCAA uptake occurs in skeletal muscles, where high levels of these amino acids might interfere with oxidation of fatty acids in muscles. This may also explain the increased glutamate observed in our patients with T2D. However, BCAA metabolism in muscle and its involvement in pathogenesis of T2D still needs further investigation.

A limitation of this study is the possibility that it is underpowered to detect subtle differences in metabolite changes. However, this study does not attempt to resolve the relative contribution molecules to exercise benefit or dysglycemic status, nor does it attempt to comprehensively assay alterations in the entire metabolome, instead focusing on key metabolic modules. Although we did not analyze the entire metabolome, we did assay a large number of metabolites (more than 400), which introduced a relatively heavy burden for multiple testing correction. It is possible in future studies to limit the scope of these analyses to metabolites with known *a priori* biological function, which would decrease the burden of study-wise significance corrections and thus decrease the probability of false negative findings. Another important limitation of the study is the medication of patients in the T2D group. All subjects were treated with metformin. Studies have shown that metformin may blunt effects of exercise training on insulin sensitivity improvements and contribute to inter-individual variability for glycemic responses to exercise (55, 56). Moreover, metformin abrogates the exercise‐mediated increase in skeletal muscle mitochondrial respiration (57). All of this should be considered when drawing conclusions on the effect of exercise intervention on skeletal muscle metabolites in T2D subjects, as this effect might be highly affected by the metformin treatment. Further studies involving T2D patients with and without metformin treatment are needed to understand how these responses are a function of metformin administration. Additionally, it is crucial to highlight, especially in the context of alterations in lipid metabolites, that the observed differences between groups and under the exercise, could be caused by the fiber type composition of muscle tissue and myocyte mitochondrial content. Studies have shown that both these factors are highly related to lipid composition of the tissue, and may play an important role in the development of insulin resistance and response to exercise interventions (28, 58). Further studies are needed to differentiate among tissue composition, where metabolite concentrations are studied in conjunction with mitochondrial content and fiber types.

Finally, in this study, only male subjects participated in the exercise intervention, and thus the observed effects should be interpreted with caution that it describes only male subjects. Additional studies are needed to explore if differences between sexes in metabolites concentrations exist in patients with dysglycemia.

In conclusion, in our study, using a metabolomic approach to explore metabolite concentration in skeletal muscle between patients at subsequent stages of dysglycemia and its response to exercise, we detected differences in several specific, clinically relevant classes of metabolites. This approach provides an opportunity to “prioritize” which metabolites should be the focus in early intervention of type 2 diabetes. We show that several specific lipid species, including particular ceramides, phosphatidylcholines and sphingomyelins, might be predominant molecules involved in these processes, confirming findings from other studies that are confined to a narrower range of lipids than ours. Of note, all have been individually found to be modifiable by exercise but have never before been simultaneously measured allowing comparisons among their relative magnitudes. Moreover, we also found that commonly unmeasured properties of amino acid metabolism might also play a crucial role in the mediation of insulin resistance development, paralleling disturbances in lipid metabolites. Together, our data suggest that the interplay between lipid and amino acid metabolism requires whole metabolomic assay and analysis, which have largely been lacking previously.

## Data Availability Statement

The raw data supporting the conclusions of this article will be made available by the authors, without undue reservation.

## Ethics Statement

The studies involving human participants were reviewed and approved by the ethics committee at the Medical University of Bialystok. The patients/participants provided their written informed consent to participate in this study.

## Author Contributions

LS, SL, and MT conceived and designed the methods, experiments, analysis, and interpretation. LS, AG, and MT, writing the manuscript, and figure preparation. LS, KS, and UP, collection and preparation of samples. UP, PK, AP, AC, PZ, and AB-Z, data collection and conduction of experiments. LS, MT, SL, UP, AP, PK, PZ, and AB-Z performed the analysis of obtained data. AK, MG, and SL, project supervision, and contribution to the interpretation of the results. All authors contributed to the article and approved the submitted version.

## Funding

This research was funded by a research grant from the Leading National Research Center KNOW (Grant ID: 56/KNOW/15).

## Conflict of Interest

The authors declare that the research was conducted in the absence of any commercial or financial relationships that could be construed as a potential conflict of interest.

## Publisher’s Note

All claims expressed in this article are solely those of the authors and do not necessarily represent those of their affiliated organizations, or those of the publisher, the editors and the reviewers. Any product that may be evaluated in this article, or claim that may be made by its manufacturer, is not guaranteed or endorsed by the publisher.
