# Supplementary material for: Metabolomic Profile of Skeletal Muscle and Its Change Under a Mixed-Mode Exercise Intervention in Progressively Dysglycemic Subjects
Source: Front Endocrinol (Lausanne). 2021 Dec 6;12:778442. doi: 10.3389/fendo.2021.778442 (PMC8685540; doi:10.3389/fendo.2021.778442)
Supplement: Supplementary file 1 [file DataSheet_1.docx]

Supplementary Material

**Supplementary Table 1. List of Metabolites measured using the AbsoluteIDQ® p400 HR Kit.**

| **Amino Acids** | | | | | |
| --- | --- | --- | --- | --- | --- |
| Ala | Alanine | | Lys | Lysine | |
| Arg | Arginine | | Met | Methionine | |
| Asn | Asparagine | | Orn | Ornithine | |
| Asp | Aspartate | | Phe | Phenylalanine | |
| Cit | Citrulline | | Pro | Proline | |
| Glu | Glutamate | | Ser | Serine | |
| Gln | Glutamine | | Thr | Threonine | |
| Gly | Glycine | | Trp | Tryptophan | |
| His | Histidine | | Tyr | Tyrosine | |
| Ile | Isoleucine* | | Val | Valine | |
| xLeu | Leucine + Isoleucine | | |  | |
| **Biogenic Amines** | | | | | |
| AcOrn | Acetylornithine | | Nitro-Tyr | Nitrotyrosine | |
| ADMA | Asymmetric dimethylarginine | | PEA | Phenylethylamine | |
| alpha-AAA | alpha-Aminoadipic acid | | Putrescine | Putrescine | |
| Carnosine | Carnosine | | Sarcosine | Sarcosine | |
| c4-OH-Pro | *cis-4-Hydroxyproline* | | SDMA | Symmetric dimethylarginine | |
| Creatinine | Creatinine | | Serotonin | Serotonin | |
| DOPA | Dihydroxyphenylalanine | | Spermidine | Spermidine | |
| Dopamine | Dopamine | | Spermine | Spermine | |
| Histamine | Histamine | | Taurine | Taurine | |
| Kynurenine | Kynurenine | | t4-OH-Pro | *trans*-4-Hydroxyproline | |
| Met-SO | Methionine sulfoxide | | |  | |
| **Acylcarnitines** | | | | | |
| AC(0:0) | Carnitine | | AC(4:1-DC) | Fumarylcarnitine | |
| AC(2:0) | Acetylcarnitine | | AC(5:0) | Valerylcarnitine | |
| AC(3:0) | Propionylcarnitine | | AC(5:0-DC) | Glutarylcarnitine | |
| AC(3:0-DC) | Malonylcarnitine | | AC(5:0-OH) | Hydroxyvalerylcarnitine | |
| AC(3:0-OH) | Hydroxypropionylcarnitine | | AC(5:1) | Tiglylcarnitine | |
| AC(3:1) | Propenoylcarnitine | | AC(5:1-DC) | Glutaconylcarnitine | |
| AC(4:0) | Butyrylcarnitine | | AC(6:0) | Hexanoylcarnitine | |
| AC(4:0-DC) | Methylmalonylcarnitine | | AC(6:0-DC) | Adipoylcarnitine | |
| AC(4:0-OH) | Hydroxybutyrylcarnitine | | AC(6:0-OH) | Hydroxyhexanoylcarnitine | |
| AC(4:1) | Butenylcarnitine | | AC(6:1) | Hexenoylcarnitine | |
| AC(7:0) | Heptanoylcarnitine | | AC(14:1-DC) | Carboxytridecenoylcarnitine | |
| AC(7:0-DC) | Pimeloylcarnitine | | AC(14:1-OH) | Hydroxytetradecenoylcarnitine | |
| AC(8:0) | Octanoylcarnitine | | AC(14:2) | Tetradecadienoylcarnitine | |
| AC(8:1) | Octenoylcarnitine | | AC(14:2-OH) | Hydroxytetradecadienoylcarnitine | |
| AC(8:1-OH) | Hydroxyoctenoylcarnitine | | AC(15:0) | Pentadecanoylcarnitine | |
| AC(9:0) | Nonaylcarnitine | | AC(16:0) | Hexadecanoylcarnitine | |
| AC(10:0) | Decanoylcarnitine | | AC(16:0-OH) | Hydroxyhexadecanoylcarnitine | |
| AC(10:1) | Decenoylcarnitine | | AC(16:1) | Hexadecenoylcarnitine | |
| AC(10:2) | Decadienoylcarnitine | | AC(16:1-OH) | Hydroxyhexadecenoylcarnitine | |
| AC(10:3) | Decatrienoylcarnitine | | AC(16:2) | Hexadecadienoylcarnitine | |
| AC(11:0) | Dimethylnonanoylcarnitine | | AC(16:2-OH) | Hydroxyhexadecadienoylcarnitine | |
| AC(12:0) | Dodecanoylcarnitine | | AC(17:0) | Heptadecanoylcarnitine | |
| AC(12:0-DC) | Dodecanedioylcarnitine | | AC(18:0) | Octadecanoylcarnitine | |
| AC(12:1) | Dodecenoylcarnitine | | AC(18:1) | Octadecenoylcarnitine | |
| AC(13:0) | Tridecanoylcarnitine | | AC(18:1-OH) | Hydroxyoctadecenoylcarnitine | |
| AC(14:0) | Tetradecanoylcarnitine | | AC(18:2) | Octadecadienylcarnitine | |
| AC(14:0-OH) | Hydroxymyristoylcarnitine | | AC(19:0) | Nonadecanoylcarnitine | |
| AC(14:1) | Tetradecenoylcarnitine | | |  | |
| **Diglycerides** | | | | | |
| DG(32:1) | | DG(36:3) | DG(41:1) | | DG-O(32:2) |
| DG(32:2) | | DG(36:4) | DG(42:0) | | DG-O(34:1) |
| DG(34:1) | | DG(38:0) | DG(42:1) | | DG-O(36:4) |
| DG(34:3) | | DG(38:5) | DG(42:2) | |  |
| DG(36:2) | | DG(39:0) | DG(44:3) | |  |
| **Triglycerides** | | | | | |
| TG(44:1) | | TG(50:3) | TG(52:6) | | TG(54:7) |
| TG(44:2) | | TG(50:4) | TG(52:7) | | TG(55:6) |
| TG(44:4) | | TG(51:1) | TG(53:3) | | TG(55:7) |
| TG(46:2) | | TG(51:2) | TG(53:4) | | TG(55:8) |
| TG(48:1) | | TG(51:3) | TG(53:5) | | TG(55:9) |
| TG(48:2) | | TG(51:4) | TG(53:6) | | TG(56:6) |
| TG(48:3) | | TG(51:5) | TG(54:2) | | TG(56:7) |
| TG(49:1) | | TG(52:2) | TG(54:3) | | TG(56:8) |
| TG(49:2) | | TG(52:3) | TG(54:4) | | TG(56:9) |
| TG(50:1) | | TG(52:4) | TG(54:5) | |  |
| TG(50:2) | | TG(52:5) | TG(54:6) | |  |
| **Lysophosphatidylcholines** | | | | | |
| LPC(12:0) | | LPC(17:1) | LPC(20:2) | | LPC(24:1) |
| LPC(14:0) | | LPC(18:0) | LPC(20:3) | | LPC-O(16:1) |
| LPC(15:0) | | LPC(18:1) | LPC(20:4) | | LPC-O(17:1) |
| LPC(16:0) | | LPC(18:2) | LPC(22:5) | | LPC-O(18:0) |
| LPC(16:1) | | LPC(20:0) | LPC(22:6) | | LPC-O(18:1) |
| LPC(17:0) | | LPC(20:1) | LPC(24:0) | | LPC-O(18:2) |
| **Phosphatidylcholines** | | | | | |
| PC(24:0) | | PC(36:1) | PC(41:5) | | PC-O(34:0) |
| PC(25:0) | | PC(36:2) | PC(41:8) | | PC-O(34:1) |
| PC(26:0) | | PC(36:3) | PC(42:0) | | PC-O(34:2) |
| PC(27:0) | | PC(36:4) | PC(42:1) | | PC-O(34:3) |
| PC(27:1) | | PC(36:5) | PC(42:2) | | PC-O(34:4) |
| PC(28:1) | | PC(36:6) | PC(42:3) | | PC-O(35:3) |
| PC(29:0) | | PC(37:0) | PC(42:4) | | PC-O(35:4) |
| PC(29:1) | | PC(37:1) | PC(42:5) | | PC-O(36:0) |
| PC(29:2) | | PC(37:2) | PC(42:6) | | PC-O(36:1) |
| PC(30:0) | | PC(37:3) | PC(42:7) | | PC-O(36:2) |
| PC(30:1) | | PC(37:4) | PC(42:10) | | PC-O(36:3) |
| PC(30:2) | | PC(37:5) | PC(43:2) | | PC-O(36:4) |
| PC(30:3) | | PC(37:6) | PC(43:6) | | PC-O(36:5) |
| PC(31:0) | | PC(37:7) | PC(44:1) | | PC-O(36:6) |
| PC(31:1) | | PC(38:0) | PC(44:3) | | PC-O(37:6) |
| PC(31:2) | | PC(38:1) | PC(44:5) | | PC-O(37:7) |
| PC(31:3) | | PC(38:2) | PC(44:6) | | PC-O(38:0) |
| PC(32:0) | | PC(38:3) | PC(44:7) | | PC-O(38:1) |
| PC(32:1) | | PC(38:4) | PC(44:10) | | PC-O(38:2) |
| PC(32:2) | | PC(38:5) | PC(44:12) | | PC-O(38:3) |
| PC(32:3) | | PC(38:6) | PC(46:1) | | PC-O(38:4) |
| PC(32:4) | | PC(38:7) | PC(46:2) | | PC-O(38:5) |
| PC(32:5) | | PC(39:0) | PC-O(26:0) | | PC-O(38:6) |
| PC(32:6) | | PC(39:1) | PC-O(26:1) | | PC-O(40:0) |
| PC(33:0) | | PC(39:2) | PC-O(28:0) | | PC-O(40:1) |
| PC(33:1) | | PC(39:3) | PC-O(28:1) | | PC-O(40:2) |
| PC(33:2) | | PC(39:4) | PC-O(29:0) | | PC-O(40:3) |
| PC(33:3) | | PC(39:5) | PC-O(30:0) | | PC-O(40:4) |
| PC(33:4) | | PC(39:6) | PC-O(30:1) | | PC-O(40:5) |
| PC(33:5) | | PC(39:7) | PC-O(30:2) | | PC-O(40:6) |
| PC(34:0) | | PC(40:1) | PC-O(31:0) | | PC-O(40:7) |
| PC(34:1) | | PC(40:2) | PC-O(31:1) | | PC-O(40:8) |
| PC(34:2) | | PC(40:3) | PC-O(31:3) | | PC-O(42:0) |
| PC(34:3) | | PC(40:4) | PC-O(32:0) | | PC-O(42:1) |
| PC(34:4) | | PC(40:5) | PC-O(32:1) | | PC-O(42:2) |
| PC(34:5) | | PC(40:6) | PC-O(32:2) | | PC-O(42:3) |
| PC(35:0) | | PC(40:7) | PC-O(32:3) | | PC-O(42:4) |
| PC(35:1) | | PC(40:8) | PC-O(33:0) | | PC-O(42:5) |
| PC(35:2) | | PC(40:9) | PC-O(33:1) | | PC-O(42:6) |
| PC(35:3) | | PC(41:1) | PC-O(33:2) | | PC-O(44:3) |
| PC(35:4) | | PC(41:2) | PC-O(33:3) | | PC-O(44:4) |
| PC(35:5) | | PC(41:3) | PC-O(33:4) | | PC-O(44:5) |
| PC(36:0) | | PC(41:4) | PC-O(33:6) | | PC-O(44:6) |
| **Sphingomyelins** | | | | | |
| SM(30:1) | | SM(34:2) | SM(38:3) | | SM(42:1) |
| SM(31:0) | | SM(35:1) | SM(39:1) | | SM(42:2) |
| SM(31:1) | | SM(36:0) | SM(39:2) | | SM(42:3) |
| SM(32:1) | | SM(36:1) | SM(40:1) | | SM(43:1) |
| SM(32:2) | | SM(36:2) | SM(40:2) | | SM(43:2) |
| SM(33:1) | | SM(37:1) | SM(40:4) | | SM(44:1) |
| SM(33:2) | | SM(38:1) | SM(41:1) | | SM(44:2) |
| SM(34:1) | | SM(38:2) | SM(41:2) | |  |
| **Cholesteryl Esters** | | | | | |
| CE(16:0) | | CE(17:2) | CE(19:2) | | CE(22:5) |
| CE(16:1) | | CE(18:1) | CE(19:3) | | CE(22:6) |
| CE(17:0) | | CE(18:2) | CE(20:4) | |  |
| CE(17:1) | | CE(18:3) | CE(20:5) | |  |

**Supplementary Table 2: Clinical characteristics of studied groups, before the initiation and after the termination of the exercise intervention. Presented are mean values with 95% confidence intervals (95%CI).**

|  | **NORMOGLYCEMIA** | | **PREDIABETES** | | **T2D** | | **p-values** | | |
| --- | --- | --- | --- | --- | --- | --- | --- | --- | --- |
| **Parameter** | **Before** | **After** | **Before** | **After** | **Before** | **After** | **Before** | **After** | **Time** |
| **Weight (kg)** | 83.80  (77.99-89.61) | 84.11  (74.21-91.03) | 101.19  (94.39-108.00) | 97.76  (89.22-103.45) | 88.89  (79.36-98.42) | 88.54  (78.89-97.31) | ***0.001*** | ***0.008*** | 0.698 |
| **HbA1c (%)** | 5.26  (5.04-5.47) | 5.13  (4.94-5.32) | 5.69  (5.48-5.91) | 5.53  (5.37-5.68) | 5.99  (5.58-6.4) | 5.92  (5.62-6.22) | ***0.001*** | ***<0.001*** | ***0.008*** |
| **Fasting Glucose (mg/dl)** | 95.73  (91.04-100.41) | 98.09  (94.62-101.56) | 119.38  (112.13-126.64) | 113.07  (108.96-117.18) | 124.13  (113.78-134.47) | 123.62  (114.3-132.94) | ***<0.001*** | ***<0.001*** | 0.948 |
| **2-h Glucose (mg/dl)** | 92.73  (75.59-109.86) | 93.36  (76.26-110.45) | 161.23  (147.61-174.85) | 126.53  (112.42-140.65) | 233.25  (180.63-285.87) | 213.50  (172.64-254.35) | ***<0.001*** | ***<0.001*** | ***0.014*** |
| **Fasting Insulin (μU/mL)** | 9.89  (7.41-12.37) | 8.89  (6.12-11.66) | 23.37  (16.78-29.97) | 23.16  (16.7-29.62) | 12.34  (8.32-16.35) | 10.93  (8.72-13.14) | ***<0.001*** | ***<0.001*** | 0.390 |
| **HOMA-b (%)** | 111.68  (82.67-140.7) | 92.65  (63.94-121.36) | 153.57  (110.13-197) | 173.80  (113.75-233.85) | 77.17  (41.91-112.43) | 65.07  (55.26-74.88) | ***0.017*** | ***<0.001*** | 0.524 |
| **HOMA-IR** | 2.35  (1.73-2.96) | 2.15  (1.48-2.83) | 6.94  (4.86-9.03) | 6.41  (4.75-8.06) | 3.77  (2.53-5.01) | 3.40  (2.49-4.31) | ***<0.001*** | ***<0.001*** | 0.261 |
| **BMI (kg/m^2^)** | 25.69  (24.19-27.2) | 25.78  (24.17-27.38) | 32.88  (30.41-35.34) | 32.73  (30.52-34.94) | 29.68  (26.62-32.74) | 29.23  (27.16-31.31) | ***<0.001*** | ***<0.001*** | 0.395 |
| **Fat mass (kg)** | 23.37  (19.05-27.7) | 22.76  (18.31-27.2) | 35.53  (31.89-39.17) | 33.49  (29.97-37.01) | 27.54  (21.32-33.76) | 25.63  (21.65-29.6) | ***<0.001*** | ***0.001*** | ***<0.001*** |
| **Lean mass (kg)** | 57.15  (53.57-60.73) | 57.84  (54.5-61.18) | 62.24  (58.24-66.25) | 63.51  (60.01-67) | 58.27  (53.26-63.29) | 59.04  (54.92-63.15) | 0.115 | 0.056 | ***0.001*** |
| **VAT mass (kg)** | 1.35  (0.9-1.8) | 1.30  (0.89-1.7) | 2.86  (2.3-3.41) | 2.71  (2.2-3.21) | 2.45  (1.57-3.33) | 2.22  (1.64-2.8) | ***0.001*** | ***0.001*** | ***0.011*** |
| **Total Chol. (mg/dl)** | 214.82  (187.42-242.22) | 201.18  (177.62-224.74) | 209.69  (193.67-225.72) | 214.53  (196-233.07) | 195.75  (158.33-233.17) | 179.37  (144.64-214.1) | 0.53 | 0.172 | 0.207 |
| **TG (mg/dl)** | 103.55  (70.88-136.21) | 91.36  (70.08-112.64) | 152.85  (123.28-182.41) | 154.01  (120.2-187.79) | 137.25  (110.96-163.54) | 130.37  (99.41-161.33) | ***0.040*** | ***0.018*** | 0.452 |
| **HDL (mg/dl)** | 61.36  (51.48-71.25) | 59.36  (51.29-67.42) | 52.26  (43.88-60.12) | 53.84  (46.32-61.37) | 56.25  (48.84-63.66) | 51.52  (43.93-59.06) | 0.229 | 0.402 | 0.349 |
| **LDL (mg/dl)** | 142.05  (115.76-168.34) | 132.02  (109.24-154.75) | 139.99  (124.75-155.22) | 145.08  (130.88-159.28) | 124.78  (89.64-159.92) | 110.61  (81.32-139.89) | 0.528 | 0.108 | 0.223 |
| **VO2max (ml/kg/min)** | 33.69  (29.76-37.62) | 34.26  (31.53-36.98) | 27.56  (26.07-29.06) | 29.69  (28.01-31.37) | 26.86  (23.34-30.38) | 29.53  (26.84-32.22) | ***0.002*** | ***0.013*** | ***0.003*** |
| **Matsuda index** | 4.73  (3.69-5.77) | 5.56  (4.13-6.99) | 1.64  (1.08-2.19) | 1.90  (1.23-2.57) | 2.95  (1.5-4.4) | 3.12  (2.29-3.96) | ***<0.001*** | ***<0.001*** | 0.092 |

**Supplementary Table 3: Concentrations of measured ACs (before the initiation and after the termination of the exercise intervention), that were significantly altered in all studied groups. Presented are mean values with 95% confidence intervals (95%CI).**

|  | **NORMOGLYCEMIA** | | **PREDIABETES** | | **T2D** | |
| --- | --- | --- | --- | --- | --- | --- |
| **Metabolite** | **Before** | **After** | **Before** | **After** | **Before** | **After** |
| **Propionylcarnitine** | 1.29 (0.87-1.71) | 1.11 (0.9-1.32) | 1.29 (1.04-1.55) | 1.1 (0.94-1.27) | 1.47 (1.19-1.75) | 0.95 (0.8-1.11) |
| **Valerylcarnitine** | 0.57 (0.31-0.84) | 0.44 (0.25-0.64) | 0.59 (0.4-0.78) | 0.46 (0.36-0.55) | 0.55 (0.33-0.77) | 0.22 (0.18-0.26) |
| **Hydroxyvalerylcarnitine** | 0.17 (0.1-0.23) | 0.12 (0.07-0.17) | 0.09 (0.06-0.12) | 0.07 (0.05-0.09) | 0.23 (0.1-0.35) | 0.09 (0.04-0.14) |
| **Malonylcarnitine** | 0.02 (0.01-0.04) | 0.03 (0.02-0.04) | 0.02 (0.01-0.02) | 0.03 (0.02-0.03) | 0.02 (0.02-0.03) | 0.03 (0.02-0.04) |
| **Hydroxyhexanoylcarnitine** | 0.09 (0.06-0.12) | 0.12 (0.08-0.17) | 0.07 (0.05-0.08) | 0.11 (0.09-0.13) | 0.09 (0.07-0.11) | 0.1 (0.07-0.12) |
| **Decanoylcarnitine** | 0.22 (0.15-0.3) | 0.38 (0.21-0.56) | 0.3 (0.2-0.41) | 0.41 (0.23-0.58) | 0.32 (0.21-0.44) | 0.36 (0.24-0.48) |
| **Decenoylcarnitine** | 0.05 (0.03-0.06) | 0.06 (0.04-0.08) | 0.06 (0.04-0.08) | 0.07 (0.05-0.1) | 0.06 (0.05-0.08) | 0.08 (0.05-0.11) |
| **Dodecanoylcarnitine** | 1.1 (0.52-1.68) | 1.88 (1.02-2.73) | 0.93 (0.74-1.13) | 1.37 (1.05-1.69) | 0.57 (0.47-0.67) | 0.65 (0.48-0.83) |
| **Dodecenoylcarnitine** | 0.23 (0.14-0.32) | 0.35 (0.25-0.44) | 0.26 (0.19-0.33) | 0.33 (0.26-0.4) | 0.28 (0.21-0.35) | 0.3 (0.24-0.37) |
| **Tridecanoylcarnitine** | 0.04 (0.02-0.06) | 0.06 (0.04-0.09) | 0.04 (0.03-0.05) | 0.05 (0.04-0.05) | 0.03 (0.02-0.03) | 0.03 (0.01-0.04) |
| **Tetradecanoylcarnitine** | 1.96 (1.14-2.78) | 3.31 (2.23-4.39) | 1.79 (1.44-2.14) | 2.53 (2.04-3.03) | 0.99 (0.82-1.15) | 1.2 (0.91-1.5) |
| **Tetradecenoylcarnitine** | 1.84 (1-2.69) | 2.68 (1.94-3.41) | 1.63 (1.27-2) | 2.31 (1.77-2.85) | 1.24 (1.01-1.46) | 1.54 (1.21-1.86) |
| **Tetradecadienoylcarnitine** | 0.32 (0.17-0.48) | 0.42 (0.29-0.55) | 0.26 (0.2-0.32) | 0.38 (0.29-0.46) | 0.23 (0.18-0.27) | 0.3 (0.22-0.39) |
| **Pentadecanoylcarnitine** | 0.22 (0.14-0.3) | 0.33 (0.23-0.43) | 0.23 (0.19-0.27) | 0.26 (0.22-0.31) | 0.09 (0.07-0.11) | 0.1 (0.08-0.13) |
| **Hexadecanoylcarnitine** | 8.72 (5.22-12.22) | 14.97 (10.61-19.34) | 9.03 (7.33-10.73) | 12.11 (9.37-14.85) | 4.67 (4.03-5.31) | 5.46 (4.64-6.28) |
| **Hexadecenoylcarnitine** | 3.09 (1.82-4.36) | 5.34 (3.9-6.78) | 2.82 (2.24-3.4) | 4.08 (3.05-5.1) | 1.36 (1.16-1.57) | 1.72 (1.42-2.02) |
| **Hexadecadienoylcarnitine** | 0.52 (0.27-0.77) | 0.75 (0.56-0.94) | 0.35 (0.27-0.44) | 0.54 (0.42-0.67) | 0.17 (0.15-0.2) | 0.26 (0.19-0.33) |
| **Heptadecanoylcarnitine** | 0.21 (0.12-0.29) | 0.35 (0.24-0.45) | 0.21 (0.17-0.25) | 0.27 (0.22-0.31) | 0.11 (0.09-0.12) | 0.13 (0.1-0.16) |
| **Octadecenoylcarnitine** | 28.96 (16.01-41.92) | 55.86 (37.97-73.75) | 29.19 (23.25-35.13) | 37.36 (28.85-45.88) | 12.2 (11.12-13.27) | 14.42 (12.98-15.86) |
| **Octadecadienylcarnitine** | 8.82 (4.57-13.07) | 14.87 (10.05-19.69) | 7.52 (5.93-9.11) | 9.82 (7.82-11.82) | 3.09 (2.68-3.49) | 3.93 (3.25-4.61) |

**Supplementary Table 4: Concentrations of measured ceramides (before the initiation and after the termination of the exercise intervention), that were significantly altered in all studied groups. Presented are mean values with 95% confidence intervals (95%CI).**

|  | **NORMOGLYCEMIA** | | **PREDIABETES** | | **T2D** | |
| --- | --- | --- | --- | --- | --- | --- |
| **Metabolite** | **Before** | **After** | **Before** | **After** | **Before** | **After** |
| **Sph** | 0.59 (0.48-0.71) | 0.48 (0.4-0.56) | 0.61 (0.5-0.72) | 0.51 (0.44-0.57) | 0.57 (0.5-0.65) | 0.46 (0.42-0.51) |
| **SPA** | 0.08 (0.06-0.1) | 0.07 (0.05-0.09) | 0.1 (0.08-0.11) | 0.06 (0.05-0.08) | 0.08 (0.07-0.1) | 0.06 (0.05-0.07) |
| **C16:0-Cer** | 0.58 (0.45-0.7) | 0.43 (0.36-0.5) | 0.55 (0.47-0.62) | 0.47 (0.41-0.54) | 0.59 (0.44-0.73) | 0.48 (0.41-0.55) |
| **C18:1-Cer** | 0.06 (0.04-0.07) | 0.04 (0.03-0.05) | 0.05 (0.04-0.07) | 0.04 (0.03-0.06) | 0.04 (0.03-0.05) | 0.03 (0.02-0.04) |
| **C18:0-Cer** | 2.02 (1.84-2.2) | 1.54 (1.41-1.68) | 2.26 (1.94-2.58) | 1.75 (1.53-1.97) | 2.19 (1.95-2.44) | 1.64 (1.35-1.93) |
| **C20:0-Cer** | 0.08 (0.07-0.09) | 0.06 (0.05-0.07) | 0.07 (0.06-0.08) | 0.06 (0.05-0.07) | 0.07 (0.06-0.09) | 0.06 (0.05-0.08) |
| **C24:1-Cer** | 2.55 (2.2-2.91) | 2.11 (1.73-2.5) | 2.65 (2.31-2.98) | 2.36 (2.02-2.7) | 2.86 (2.67-3.04) | 2.55 (2.11-2.99) |
| **C24:0-Cer** | 1.63 (1.27-1.99) | 1.19 (1.04-1.35) | 1.28 (1.06-1.51) | 1.13 (0.96-1.3) | 1.24 (1.05-1.44) | 1.1 (0.96-1.25) |
| **Total Cer** | 8.07 (7.07-9.07) | 6.3 (5.61-7) | 7.8 (6.81-8.78) | 6.76 (6.14-7.37) | 7.86 (7.29-8.44) | 6.78 (6.08-7.48) |
